# Ambulatory Health Care Service Use and Costs Among Commercially Insured US Adults With Congenital Heart Disease

**DOI:** 10.1001/jamanetworkopen.2020.18752

**Published:** 2020-09-24

**Authors:** Anushree Agarwal, Eric Vittinghoff, Janet J. Myers, R. Adams Dudley, Abigail Khan, Anitha John, Gregory M. Marcus

**Affiliations:** 1 Division of Cardiology, Department of Medicine, University of California, San Francisco; 2 Department of Epidemiology and Biostatistics, University of California, San Francisco; 3 Division of Prevention Science, Department of Medicine, University of California, San Francisco; 4 Department of Medicine, Philip R. Lee Institute for Health Policy Studies, School of Medicine, and Center for Healthcare Value, University of California, San Francisco; 5 Adult Congenital Heart Disease Program, Knight Cardiovascular Institute, Oregon Health & Science University, Portland; 6 Division of Cardiology, Children's National Health System, Washington, DC

## Abstract

This study examines the ambulatory health care use and costs of adults with congenital heart disease in the US.

## Introduction

Adults with congenital heart disease (CHD) are a rapidly increasing population,^1^ have many comorbidities,^2^ and require frequent monitoring.^3^ However, little is known about their ambulatory health care use and associated costs in the US.

## Methods

*International Statistical Classification of Diseases and Related Health Problems, 10th Revision (ICD-10)* codes were used to identify patients with CHD, as described previously (eTable in the Supplement).^2^ The control group was selected from a random sample of age- and sex-matched individuals without CHD and with at least 1 year of data equivalent to that of the patients with CHD. Comorbidities were identified with Elixhauser comorbidity measures.^3^ The American Heart Association/American College of Cardiology anatomic classification was used to categorize adults with CHD as having simple, moderately complex, and complex disease.^4^

Wilcoxon and χ^2^ tests were used for comparisons of continuous and categoric variables, respectively. To estimate the independent associations of age, sex, US region, beneficiary status, comorbidities, and lesion type with costs, we used zero-inflated negative binomial models. These models accommodate the severe right skewing of costs, as well as the excess of observations with no costs, relative to the standard negative binomial distribution. Adjusted mean costs by lesion group were obtained by regression standardization, based on the fitted negative binomial models. Two-tailed *P* < .05 was considered statistically significant. Analyses were performed with Stata version 16.0. Data were analyzed on January 28, 2020.

## Results

The mean (SD) age of 33 892 patients included in the study cohort was 35.2 (14.2) years, and 48.8% were women. Of 16 946 patients, 5168 (30.5%) had complex CHD, 5719 (33.8%) had moderately complex CHD, and 6059 (35.7%) had simple CHD.

Compared with individuals without CHD, those with CHD had more comorbidities, more health care visits, and higher expenditures (Table). After multivariate adjustments, ambulatory costs remained significantly higher for all types of adults with CHD than for those without it (Figure).

**Table.** Comparison of Baseline Characteristics, Health Care Use, and Costs for Adults With vs Without Congenital Heart Disease, 2016

Characteristics	Median (IQR)		*P* value
	ACHD (n = 16 946)	Non-ACHD (n = 16 946)	
Age, mean (SD), y	35.2 (14.2)	35.2 (14.2)	>.99
Female sex, No. (%)	8275 (48.8)	8275 (48.8)	>.99
Primary beneficiaries, No. (%)	7721 (47.5)	7799 (53.2)	<.001
US region, No. (%)			
Northeast	3579 (22.0)	2561 (17.5)	<.001
North Central	3434 (21.1)	3071 (21.0)	
South	6894 (42.4)	6656 (45.4)	
West	2321 (14.3)	2313 (15.8)	
Unknown	42 (0.3)	48 (0.3)	
Comorbidities, No. (%)^a^			
Any comorbidity	11 547 (77.5)	9230 (62.0)	<.001
Cardiovascular	930 (6.2)	68 (0.5)	<.001
Noncardiovascular	3964 (26.6)	2556 (17.2)	<.001
Services^b^			
Physician outpatient visits	6.0 (3.0-12.0)	3.0 (1.0-7.0)	<.001
Primary care	2.0 (1.0-4.0)	1.0 (0.0-3.0)	<.001
Cardiologists	1.0 (0.0-2.0)	0.0 (0.0-0.0)	<.001
Other specialists	0.0 (0.0-1.0)	0.0 (0.0-1.0)	<.001
Nonphysician outpatient visits	1.0 (0.0-4.0)	0.0 (0.0-2.0)	<.001
Emergency department visits	0.0 (0.0-2.0)^c^	0.0 (0.0-1.0)^c^	<.001
Prescription drug claims	8.0 (1.0-18.0)	3.0 (0.0-11.0)	<.001
Expenditures, $^d^			
Total cost			
Ambulatory	3598 (1221-9454)	1068 (230-3640)	<.001
Physician	1120 (440-2503)	375 (69-1083)	<.001
Nonphysician	839 (90-3413)	125 (0-704)	<.001
Emergency department cost	2005 (993-4035)	1583 (808-3209)	<.001
Prescription drug cost	213 (13-1237)	64 (0-527)	<.001
Out-of-pocket ambulatory cost	802 (246-1862)	261 (33-892)	<.001

Abbreviations: ACHD, adults with congenital heart disease; IQR, interquartile range.

^a^ Cardiovascular comorbidities include congestive heart failure, arrhythmias, pulmonary circulation disorders, hypertension, hypercholesterolemia, coronary artery disease, peripheral vascular disorders, and stroke. Noncardiovascular comorbidities include diabetes, obesity, neurologic disorder, hypothyroidism, liver disease, peptic ulcer, AIDS, any tumor, rheumatoid arthritis/collagen vascular disease, coagulopathy, weight loss, fluid and electrolyte disorders, anemia, kidney disease, substance abuse, psychiatric disorder, and chronic pulmonary disease.

^b^ Other specialists include neurologist, endocrinologist, gastroenterologist, hematologist, infectious disease specialist, nephrologist, pulmonologist, rheumatologist, gynecologist, psychiatrist, and oncologist. These specialists were chosen because patients with congenital heart disease are known to have a higher incidence of noncardiac comorbidities that require management by these specialists.^2^ Nonphysician visits include those for diagnostic testing, physical therapist, etc. We included only emergency department visits that did not result in an inpatient admission. Prescription drug claims represent the number of prescriptions filled by the beneficiary during the given period.

^c^ Values for emergency department visits represent median (IQR).

^d^ Total ambulatory cost includes the combination of total outpatient, physician outpatient, nonphysician outpatient, emergency department, and prescription drug costs. Total out-of-pocket costs include copayments, deductibles, and payments for services not covered by insurance. Out-of-pocket costs were counted as a component of the total ambulatory costs. Emergency department cost represents expenditures only for patients who had any emergency department visit.

**Figure.**
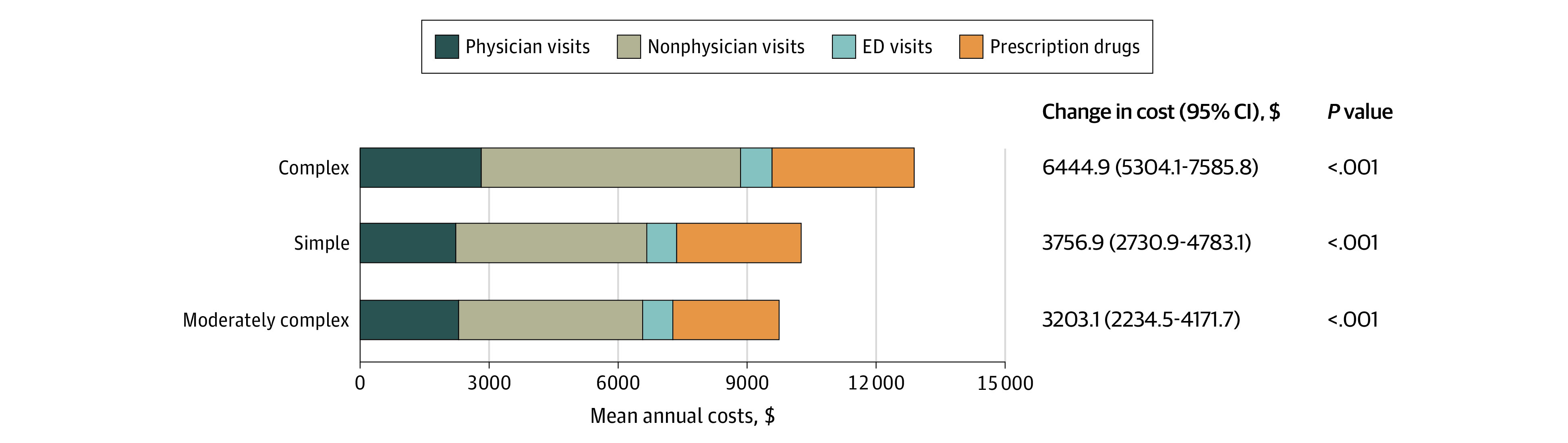
Adjusted Annual Total Ambulatory Costs and Out-of-Pocket Costs Horizontal bars show each component of ambulatory health care cost (1a) and out-of-pocket cost (1b) for adults with congenital heart disease (CHD), by lesion category; the overall length of each bar indicates the total cost. Change in cost indicates the adjusted difference in overall cost compared with that for frequency-matched non-CHD patients. All costs and cost differences are adjusted for age, sex, US region, beneficiary status, and cardiac and noncardiac comorbidities. ED indicates emergency department. Lesions included within each CHD type are complex (Eisenmenger syndrome, common ventricle, hypoplastic left heart syndrome, transposition of great arteries, tetralogy of Fallot, truncus arteriosus, and endocardial cushion defect), simple (ventricular septal defect and patent ductus arteriosus), and moderately complex (Ebstein anomaly, coarctation of aorta, anomalies of the pulmonary artery, anomalies of the pulmonary valve, anomalies of the tricuspid valve, unspecified septal defects, anomalies of the great vein, subaortic stenosis, and aortic anomalies).

Among patients with CHD, after multivariate adjustments, factors independently associated with ambulatory costs were 10-year increase in age (cost ratio, 1.17; 95% CI, 1.13-1.21), female sex (cost ratio, 1.14; 95% CI, 1.05-1.23), primary beneficiary (cost ratio, 0.88; 95% CI, 0.81-0.96), complex CHD (cost ratio, 1.43; 95% CI, 1.29-1.59), cardiac comorbidities (cost ratio, 2.17; 95% CI, 1.90-2.46), and noncardiac comorbidities (cost ratio, 1.92; 95% CI, 1.75-2.10) (*P* < .005 for all).

## Discussion

Annual ambulatory health care use and costs were significantly higher for commercially insured adults with CHD than those without it, even after adjusting for their baseline characteristics and comorbidities. Among adults with CHD, complex CHD and presence of comorbidities were independently associated with the highest cost ratio magnitude. This demonstrates the extraordinary health care needs of these patients with complex disease, who usually have multisystem disease,^2^ and underscores the importance of developing structured work flows to appropriately allocate resources. Our novel CHD severity–specific health care cost estimates may help patients in their personal financial planning (selecting a health insurance plan that will minimize their financial risk, such as opting for employee-provided health savings plans) and policy makers in designing affordable and appropriate health plans.

Our study limitations include reliance on *ICD-10* codes and limited generalizability to patients who are not commercially insured. In contrast to previous studies of adults with CHD that primarily reported charges,^5,6^ our estimates are directly reflective of actual costs and therefore pertinent to understanding health resources required for these patients.

In conclusion, we provide data that could be useful to educate clinicians, health care organizations, and patients to guide resource allocation, enhance more efficient work flows, and inform realistic financial expectations.

## Notes

Supplement. **eTable.** *International Classification of Disease-10* Codes for Congenital Heart Disease (CHD) Lesions
