# Supplementary material for: Ambulatory Health Care Service Use and Costs Among Commercially Insured US Adults With Congenital Heart Disease
Source: JAMA Netw Open. 2020 Sep 24;3(9):e2018752. doi: 10.1001/jamanetworkopen.2020.18752 (PMC7516600; doi:10.1001/jamanetworkopen.2020.18752)
Supplement: Supplement. — eTable. International Classification of Disease-10 Codes for Congenital Heart Disease (CHD) Lesions [file jamanetwopen-e2018752-s001.pdf]

Supplementary Online Content

Agarwal A, Vittinghoff E, Myers JJ, et al. Ambulatory health care service use and costs among commercially insured US adults with congenital heart disease. *JAMA Netw Open*. 2020;3(9):e2018752. doi:10.1001/jamanetworkopen.2020.18752

eTable. *International Classification of Disease-10* Codes for Congenital Heart Disease (CHD) Lesions

This supplementary material has been provided by the authors to give readers additional information about their work.

© 2020 Agarwal et al. *JAMA Network Open.*

Identifying congenital heart disease (CHD) cohort:

Patients were identified as having CHD if they had a diagnosis code for any CHD lesion per International Classification of Disease, Tenth Revision (ICD-10) codes as listed below. If an ICD-9 or ICD-10 code for CHD was present on any inpatient or outpatient claim at any billing position during the period of enrollment, these patients were then considered to have CHD. For patients with codes for more than one CHD diagnosis, we used the hierarchical algorithm proposed by Broberg et al.[1] to designate one condition per patient as their principal CHD diagnosis. We excluded ICD codes that have lower specificity for CHD, including atrial septal defect, bicuspid aortic valve, aortic stenosis, and unspecified congenital anomalies. We also excluded any patients who had pregnancy or delivery related claims during the study period in order to avoid inclusion of pregnant women with fetuses affected by CHD.

**eTable. International classification of disease -10 Codes for Congenital Heart Disease (CHD) Lesions**	
Eisenmenger (CHD code AND cyanosis)	I27.83 PLUS other congenital code (I27.83 +Q20-Q28)
Hypoplastic left heart syndrome	Q23.4
Common ventricle	Q20.4
Transposition Complex	Q20.1, Q20.3, Q20.5, Q20.8
Tetralogy of Fallot	Q21.3
Truncus Arteriosus	Q20.0
Endocardial Cushion Defect	Q21.2
Ebstein's Anomaly	Q22.5
Aortic Coarctation	Q25.1
Anomalies of the Pulmonary Artery (except pulmonary atresia)	Q25.6, Q25.79, Q25.5, Q25.71
Anomalies of the Pulmonary Valve	Q22.1, Q22.2, Q22.3
Anomalies of the Tricuspid valve	Q22.4, Q22.8, Q22.9
Ventricular septal defect	Q21.0
Patent ductus arteriosus	Q25.0
Anomalies of Veins	Q26.2, Q26.3, Q26.9
Unspecified Defect of Septal Closure	Q21.9
Subaortic stenosis	Q24.4
Aortic anomalies	Q25.41, Q25.42, Q25.43, Q25.44, Q25.48, Q25.49
CHD – congenital heart disease	

[1] Broberg C, McLarry J, Mitchell J, et al. Accuracy of administrative data for detection and categorization of adult congenital heart disease patients from an electronic medical record. *Pediatr Cardiol.* 2015;36(4):719-725.

© 2020 Agarwal et al. *JAMA Network Open.*
